# Social interactions and quality of life of residents in aged care facilities: A multi-methods study

**DOI:** 10.1371/journal.pone.0273412

**Published:** 2022-08-29

**Authors:** Joyce Siette, Laura Dodds, Didi Surian, Mirela Prgomet, Adam Dunn, Johanna Westbrook

**Affiliations:** 1 Centre for Health Systems and Safety Research, Australian Institute of Health Innovation, Macquarie University, New South Wales, Australia; 2 MARCS Institute for Brain, Behaviour and Development, Western Sydney University, New South Wales, Australia; 3 Centre for Health Informatics, Australian Institute of Health Innovation, Macquarie University, New South Wales, Australia; 4 Biomedical Informatics and Digital Health, School of Medical Sciences, Faculty of Medicine and Health, The University of Sydney, New South Wales, Australia; University of Illinois at Urbana-Champaign, UNITED STATES

## Abstract

**Background:**

The relationship between social contact and quality of life is well-established within the general population. However, limited data exist about the extent of social interactions in residential aged care facilities (RACFs) providing long-term accommodation and care. We aimed to record the frequency and duration of interpersonal interactions among residents in RACFs and identify the association between residents’ interpersonal interactions and quality of life (QoL).

**Materials and methods:**

A multi-methods study, including time and motion observations and a QoL survey, was conducted between September 2019 to January 2020. Thirty-nine residents from six Australian RACFs were observed between 09:30–17:30 on weekdays. Observations included residents’ actions, location of the action, and who the resident was with during the action. At the end of the observation period, residents completed a QoL survey. The proportion of time residents spent on different actions, in which location, and with whom were calculated, and correlations between these factors and QoL were analysed.

**Results:**

A total of 312 hours of observations were conducted. Residents spent the greatest proportion of time in their own room (45.2%, 95%CI 40.7–49.8), alone (47.9%, 95%CI 43.0–52.7) and being inactive (25.6%, 95%CI 22.5–28.7). Residents were also largely engaged in interpersonal communication (20.2%, 95%CI 17.9–22.5) and self-initiated or scheduled events (20.5%, 95%CI 18.0–23.0). Residents’ interpersonal communication was most likely to occur in the common area (29.3%, 95%CI 22.9–35.7), residents’ own room (26.7%, 95%CI 21.0–32.4) or the dining room (24.6%, 95%CI 18.9–30.2), and was most likely with another resident (54.8%, 95%CI 45.7–64.2). Quality of life scores were low (median = 0.68, IQR = 0.54–0.76). Amount of time spent with other residents was positively correlated with QoL (r = 0.39, p = 0.02), whilst amount of time spent with facility staff was negatively correlated with QoL (r = -0.45, p = 0.008).

**Discussion and conclusions:**

Our findings confirm an established association between social interactions and improved QoL. Opportunities and activities which encourage residents to engage throughout the day in common facility areas can support resident wellbeing.

## Introduction

With an ageing population, the demand for long-term care such as assisted living facilities and residential aged care facilities (RACFs) (also known as care homes or nursing homes), is growing [1]. Social interactions and empathetic social support are important contributors to quality of life for older adults in RACFs [2–4]. However accumulative findings indicate that residents are found to be largely inactive, both socially and physically [5–11]. The consequences of both forms of inactivity are detrimental impacts on health and wellbeing including a decline in functional status [12], physical strength [12, 13], lower self-esteem [14], and mortality [15].

A recent review found that a low number of social ties and low reciprocity (i.e., one-way connection) are common features of residents’ social networks in RACFs. Transitioning into a RACF often means a disruption to older adults’ usual social routines, which can further reduce their social relationships and autonomy and contribute to feelings of loneliness and isolation [16]. There have been few studies investigating how residents spend their time in RACFs, in particular, to capture information about residents’ engagement with their social environment, a valid and reliable indicator of quality in RACFs [8, 17, 18].

Studies using direct observations or clinical notes have found that resident-staff interactions are infrequent, short and primarily oriented to physical care [7, 8]. Residents were often largely sedentary, have low engagement in social and physical activities and in isolation for between 40–90% of the observed periods [5, 7–9, 19–21]. One study further found that residents with higher physical dependency experienced a very small proportion (4%) of socially driven resident-staff interactions [7].

However, previous studies observing resident activities and their effect on health outcomes have often failed to comprehensively capture important dimensions of social interactions including, for instance, the size of a resident’s social networks, and frequency and duration of interactions [5, 8, 9]. Some studies have examined residents’ physical activity and treated this as a proxy for social interactions [10, 11], whilst others have focused on short observation periods (e.g., 10 minutes) and thus are unable to capture the breadth of residents’ engagement in activities at a facility [5, 7, 14]. Furthermore, methods for measuring resident activity either use clinical notes [22], technology that is susceptible to error and misinterpretation (e.g., [7, 11, 20]), or are biased by relying on the knowledge of staff [23]. Critically, most studies heavily emphasize the *physical* aspects of interactions (e.g., body positions of sitting, lying or standing), and neglect aspects of *social* engagement, such as the duration of a conversation. Therefore, the extent to which residents are socially engaged with other residents and staff in their day-to-day routines is not yet fully understood. Despite the evidence suggesting that social networks and social support can impact on quality of life, investigations into the relationship between specific social interactions and such as quality of life (QoL), remain largely unexplored in aged care.

Reliable observations that can quantify residents’ social interactions will help to inform future interventions targeting improvements in participation and wellbeing. This study thus aimed to (1) record the frequency and duration of social interactions among residents in residential care, and (2) identify the association between social interactions and the QoL of residents.

## Materials and methods

### Study design and sample

This was a multi-method study consisting of a prospective observational time and motion study, and a QoL survey, of residents from six aged care facilities in Sydney, New South Wales (NSW), Australia between 30 September 2019 and 16 January 2020. A publicly available list of residential aged care providers across NSW was used to systematically contact providers via email or phone and invite them to participate in the study. A meeting was organised with facility managers who indicated their interest in the study, after which, they were given time to consult with organisation managers and facility staff regarding participation and then signed the provider consent form. Following this, researchers created an observation schedule which was agreed upon by the facility and sought to recruit resident participants. Inclusion criteria of observed participants were (1) aged 55 years or above; (2) residing in a residential aged care facility in an independent ward; (3) willing to participate in the study; (4) capable of providing written consent; (5) no medical diagnosis of dementia or significant brain trauma; and (6) not receiving high level continuous care services and support. The RACFs housed a total of 392 residents, of which 40 residents were first identified and approached by the facility manager to seek initial interest in participating in the study (see S1 File for the participant selection flow chart). The research team approached these 40 residents, and 39 consented to participate, with one declining due to lack of interest.

### Ethical considerations

The study gained ethical approval from the Macquarie University Human Research Ethics Committee (HREC) prior to the commencement of participant recruitment (HREC 5160). Ethics processes pertaining to vulnerable populations, specifically related to the recruitment, capacity to consent and withdrawal of persons were adhered to.

### Data collection tools

#### WOMBAT

Observational data were recorded on a handheld tablet, using the Work Observation Method By Activity Timing (WOMBAT) tool [24–30]. WOMBAT is a validated time and motion application that enables observers to collect data on multidimensional aspects of work patterns and communication that is then automatically time-stamped in the field in real-time [24]. Examples of data dimensions collected include quantifying *how* and *with whom* different health and aged care professionals spend time, with the possibility of tailoring an observation template to answer a particular research question. WOMBAT has been applied in a number of contexts [25–30] including critical care to measure clinicians’ patterns of work and communication as well as interruptions and multitasking experienced by doctors and nurses during surgery [24–26, 28, 29], and in home-based aged care services to understand case managers’ work patterns [27, 30].

As this was the first time the WOMBAT tool had been applied in a residential aged care setting, the research team discussed potential resident-specific dimensions and piloted the classification system prior to data collection. This resulted in three broad dimensions which was incorporated into WOMBAT software on a tablet computer. The dimensions are described in Table 1 and are “*what actions*” (daily actions the residents engaged in, e.g., communication, walking, stationary, eating, exercising), “*where*” (locality of action, e.g., resident’s room, dining room, common room) and “*who action was completed with*” (with whom the resident was engaged with during the action, e.g., other residents, staff, alone) (see S1 File).

**Table 1:** WOMBAT dimension classification.

Dimension Category	Definition	Examples
What action	Daily actions the residents engage in.	Broad activities including *communication* (e.g., resident is having a conversation with someone or is engaging in vocalised monologue and may coincide with any other “what” actions; if communication occurs concurrently with another action then it was subsequently classified as “communication”), *walking* (i.e., resident is mobilising, with or without a mobility aid), *stationary* (i.e., resident is immobile in either sitting, standing or lying position), *eating* (resident is consuming food or drink), *sleeping*, *activities* (includes both self-initiated and scheduled activities, where self-initiated actions are ones that the resident engages in that are not part of the daily facility program/calendar or their personal care routine [e.g. drawing, reading, watching TV in room]. Scheduled actions that the resident engages in that are scheduled [e.g. doctor/specialist appointment, family outing] or are part of the daily facility program/calendar but does not include physical activity).
Where	Locations accessible to the residents where they engage in an activity.	Resident’s own room or another resident’s room, common areas, dining room or kitchen, or outside.
Who action was completed with	With whom the resident is engaging in an activity with.	Another resident, nurse, staff aid, family, allied health or no one.

The observation team consisted of four observers who received training from a researcher with extensive experience using WOMBAT (MP). The training involved explanation of the observation process and definitions for the WOMBAT template and dimensions. Training also included a practice observation with a 30-minute role-play that consisted of research members acting as residents performing daily actions. Inter-rater reliability was assessed between the four observers and one researcher (MP) who acted as a gold standard and was high (kappa >0.88 for time spent in action and >0.86 for time spent in location).

#### Quality of life

Quality of life was measured using the brief EQ-5D-5L instrument, a generic instrument consisting of a self-administered health index and has five domains including mobility, self-care, pain/discomfort, usual activities and anxiety/depression [31, 32]. The nominal range of the EQ-5D-5L index scores is 0 (poor health) to 1 (perfect health), but negative scores as low as −0.59 are possible for health states deemed to be worse than death [31, 32]. The EQ-5D-5L has demonstrated excellent convergent validity in a variety of patient groups across multiple countries, reduced ceiling effects and good discriminatory power [33]. The EQ-5D-5L is also an appropriate tool to measure quality of life in older adults in aged care [31]. Cronbach’s alpha was 0.602, which indicates a moderate level of internal consistency for this specific sample, despite the instrument consisting of multiple dimensions with single items.

### Data collection procedure

Residents were unobtrusively observed by a member of the research team who stood at least two meters away from the resident during the observation period. For each resident, the observation time was 8 hours in total, which consisted of four session of two hours blocks between 9:30 and 17:30 during weekdays. Both the period of observation and the days of observation were randomly allocated and counterbalanced amongst the four dedicated observers to ensure that hours were sampled proportionately (e.g., Resident 1 was observed on Monday at 9.30–11.30am, Tuesday at 11.30–1.30pm, Wednesday at 1.30pm-3.30pm and Thursday at 3.30pm-5.30pm, see S1 File). Residents were shadowed throughout the facility and within their rooms. Observations were not conducted in residents’ private bathrooms and this time was recorded as “non-observable”. Other non-observable observations included when activities are conducted off-site (i.e., outside of the facility grounds).

Residents provided responses to the EQ-5D-5L instrument following the end of their observation.

### Analyses

To assess residents’ activity patterns, we calculated the proportion of total observed time for each action, location, and with whom the action was with. 95% confidence intervals (CIs) for the proportion of total time were obtained using the large sample normal approximation. Pearson’s correlation coefficient (r) was used to test the strength and direction of linear relationships between quality of life scores and time spent in specific actions, time spent in different locations, and time spent with different people, controlling for gender and age. Correlations of less than 0.3 are described as small or weak, between 0.3 and 0.5 as medium or moderate, and greater than 0.5 as large or strong [32]. Statistical analysis was performed using the SPSS Version 25 (IBM, Armonk, NY, USA).

## Results

### Study population

Residents’ sociodemographic characteristics are shown in Table 2. The sample was mostly women (76.3%), with a mean age of 87.6 years (SD = 6.6). Most residents were widowed (81.6%), born in Australia (60.5%), spoke English as their main language (60.5%), had high care needs (94.7%), and did not require the use of wheelchairs (76%).

**Table 2:** Sociodemographic characteristics of residents (N = 39).

Characteristic	N (%)
**Age**	
Mean [SD]	87.6 (6.6])
71–80	14 (36.8)
81–90	17 (44.7)
≥ 90 years	7 (18.4)
**Gender**	
Female	29 (76.3)
Male	9 (23.7)
**Marital Status**	
Divorced/Single/Widow	31 (81.6)
Married	5 (13.2)
Missing	2 (5.2)
**Country of Birth**	
Australia	23 (60.5)
Ireland	1 (2.6)
Italy	14 (36.8)
**Main language**	
English	23 (60.5)
Other	15 (39.5)
**Education**	
Primary school (<7 years)	15 (39.5)
High school (<11 years)	8 (21.1)
High school (<13 years)	5 (13.2)
Tertiary level education (<17 years)	3 (7.9)
Trade/Diploma	7 (18.4)
**Care Need** ^1^	
High	36 (94.7)
Low	2 (5.3)
**Require Wheelchair**	
Yes	9 (23.7)
No	29 (76.3)

^**1**^ Residents who require almost complete assistance with most daily living activities are classified as having high care needs. This includes accommodation, meals, laundry, room cleaning, personal care and clinical care.

### Actions and time distribution

During the 312 hours of observation, a total of 4,417 actions were observed. The action-specific distribution of residents’ time is shown in Table 3. Residents spent the greatest proportion of time being stationary (25.6%), which was followed by self-initiated or scheduled activities (20.5%) and communication (20.2%).

**Table 3:** Number of observations, total and mean observation time, and percentage of total observation time of 39 residents according to three dimensions of actions.

Dimension	No. of Observations	Total action time (hours)	Mean observation time (secs)	Percentage of total observation time (%, 95% CI*)
**Where**				
Own Room	1394	144.5	373.1	45.2 (40.7–49.8)
Common Area/Lounge	985	65	237.5	20.4 (18.0–22.7)
Kitchen/Dining Room	1297	59.5	165.3	18.6 (16.5–20.8)
Other	72	19.7	985	6.1 (3.3–9.0)
Corridor	567	16	101.9	5.0 (4.3–5.8)
Outside	90	14.1	565.5	4.4 (2.4–6.5)
Other Residents Room	18	0.4	79.9	0.1 (0.1–0.2)
**What action**				
Stationary	1065	81.7	275.2	25.6 (22.5–28.7)
Scheduled or self-initiated activity	387	65.3	607.9	20.5 (18.0–23.0)
Communication	1745	57.1	132.9	20.2 (17.9–22.5)
Non-observable	200	39	702.6	12.2 (9.0–15.5)
Exercise	148	26.7	649.7	8.4 (5.9–10.8)
Walking	670	15.2	81.5	4.8 (3.9–5.6)
Sleeping	66	14.2	774.9	4.5 (2.8–6.1)
Eating	134	12.4	332.7	3.9 (3.3–4.5)
Other	2	0	70	0.01 (0–0)
**Who action was completed with**				
No One	1428	152.8	385.2	47.9 (43.0–52.7)
Other Resident	1571	110.7	253.6	34.7 (31.0–38.3)
Staff	1107	25.7	83.7	8.0 (6.5–9.7)
Family	125	16.5	474.8	5.2 (3.0–7.3)
Other	192	13.6	254.6	4.3 (2.0–5.6)

* CI = confidence interval.

Residents were observed spending most of their day in their own room (45.2%), followed by areas of communal gathering (20.4%) and food preparation or consumption (18.6%). Residents spent the greatest proportion of their time on their own (47.9%). A third of their time was with another resident (34.7%) and a small proportion of time was with staff (8.0%) or family (5.2%).

**Fig 1:**
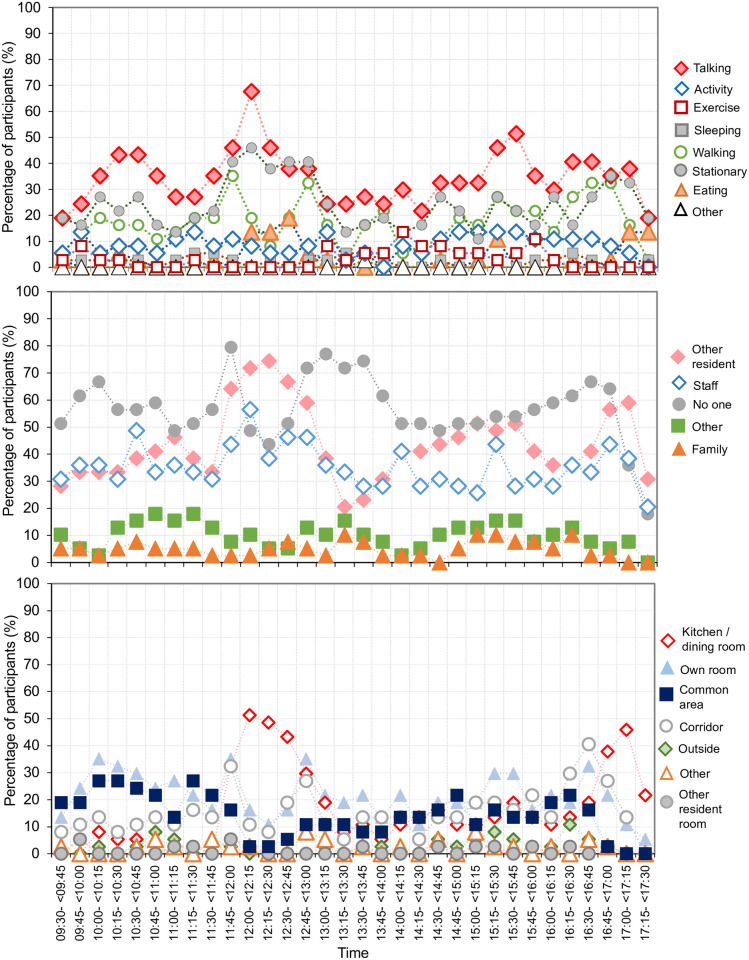
Percentage of residents (N = 39 observed over a total of 312 hours and aggregated in 15-minute period of time) engaged in a type of observed action (top panel), with whom the resident was with (middle panel) and where the resident was located within the aged care facility (bottom panel) across an 8 hour period (09:30–17:30).

Fig 1 shows what actions residents were most likely to be found doing during specific times during the day, where they spent their time and who they were most likely to be interacting with, in 15-minute intervals aggregates. Residents were observed spending most of their morning in the common area/lounge, midday and early evenings in the dining room, with an increase in observations in the residents’ own room in the afternoon. Social interactions (i.e., direct communication) peaked during meal periods, followed by increased stationary periods directly after these events. During meals, there was increased interactions among both staff aids and other residents. Outside of these times, several other actions were seen in highly fluctuating numbers.

### Actions by location and respondent

Table 4 shows the type of actions residents made in specific locations in the facility. Residents were most likely to be engaged in scheduled or self-initiated activities in their own room (75.8%) followed by in the common area (14.9%). When residents were eating, this was most likely to occur in the kitchen or dining room (60.9%), the common area (21.2%) or their own room (17.7%). When exercising, residents were most likely to be in the common area (46.3%), kitchen or dining room (20.0%) or another location which was set up for the exercise routines (18.7%). Residents who were observed as sleeping were most likely located in their own room (83.5%) or in the common area (12.3%). When residents were engaged in interpersonal communication, this was most likely to occur in either the common area (29.3%), their own room (26.7%) or the dining room (24.6%). When assessing walking, residents spent the largest proportion of time in the corridor (41.3%), followed by in their own room (30.6%) and the dining room (10.4%).

**Table 4:** Number of observations, total and mean observation time, and percentage of total observation time by resident action and its location.

What action	Where	No of observations	Total task time (hours)	Mean observation time (secs)	Percentage of total observation time (%, 95% CI*)
Activity	Common Area/Lounge	125	11.3	325.5	14.9 (11.3–18.5)
	Corridor	9	0.5	218.2	0.7 (0.2–1.2)
	Kitchen/Dining Room	29	4.2	517.3	5.5 (2.5–8.5)
	Other	4	0.6	520.8	0.8 (0–1.9)
	Other Residents’ Room	1	0.0	27.0	0.0
	Outside	11	1.8	588.3	2.4 (1.2–3.5)
	Own Room	272	57.4	760.0	75.8 (65.8–85.7)
Eating	Common Area/Lounge	19	2.6	498.3	21.2 (13.6–28.9)
	Corridor	1	0.0	22.0	0
	Kitchen/Dining Room	93	7.6	292.5	60.9 (49.7–72.4)
	Own Room	21	2.2	375.7	17.7 (9.3–26.1)
Exercise	Common Area/Lounge	105	12.8	440.2	46.3 (29.9–62.8)
	Kitchen/Dining Room	36	5.5	554.7	20.0 (11.0–29.0)
	Other	4	5.2	4667.0	18.7 (6.7–30.8)
	Outside	3	2.5	3006.3	9.0 (0–31.6)
	Own Room	17	1.6	346.2	5.9 (2.5–9.3)
Non-observable	Common Area/Lounge	8	1.0	437.8	2.0 (0–4.3)
	Kitchen/Dining Room	1	0.1	194.0	0.1
	Other	20	12.5	2242.0	26.0 (11.3–40.6)
	Outside	6	4.2	2501.0	8.7 (0–19.1)
	Own Room	197	3.0	553.7	63.3 (48.2–78.2)
Sleeping	Common Area/Lounge	12	1.8	526.0	12.3 (2.6–22.1)
	Kitchen/Dining Room	3	0.6	720.0	4.2 (0–17.0)
	Own Room	51	11.9	836.7	83.5 (48.7–100.0)
Stationary	Common Area/Lounge	226	16.2	257.7	18.0 (14.4–21.5)
	Corridor	49	1.1	82.2	1.2 (0.7–1.8)
	Kitchen/Dining Room	502	26.3	188.4	29.2 (24.5–33.8)
	Other	8	0.4	199.4	0.5 (0–1.3)
	Other Residents’ Room	1	0.0	176.0	0.1
	Outside	6	0.3	192.0	0.4 (0–0.7)
	Own Room	412	45.7	399.5	50.7 (41.0–60.5)
Communication	Common Area/Lounge	466	20.2	156.1	29.3 (22.9–35.7)
	Corridor	222	8.1	130.6	11.7 (9.0–14.4)
	Kitchen/Dining Room	639	16.9	95.5	24.6 (18.9–30.2)
	Other	27	0.9	116.9	1.3 (0.6–2.0)
	Other Residents’ Room	16	0.3	77.3	0.5 (0.3–0.7)
	Outside	37	4.1	402.4	6.0 (1.7–10.3)
	Own Room	473	18.4	140.2	26.7 (21.0–32.4)
Walking	Common Area/Lounge	78	1.5	69.3	9.6 (5.9–13.4)
	Corridor	299	6.4	77.6	41.3 (32.8–49.6)
	Kitchen/Dining Room	116	1.6	50.3	10.4 (7.4–13.3)
	Other	7	0.1	42.0	0.5 (0.4–0.7)
	Outside	27	1.2	161.5	7.8 (4.1–11.4)
	Own Room	186	4.8	92.3	30.6 (16.9–44.1)

*CI = Confidence interval.

The proportion of time engaged in a particular action and with whom is displayed in Table 5. When assessing communication, residents spent most of their time with another resident (54.8%), followed by staff (21.9%) and family (11.6%). When residents were undertaking scheduled or self-initiated activities, they were most likely to be on their own (80.8%) or with another resident (14.3%). Residents largely spent their time eating with another resident (69.9%) or on their own (17.9%). Similarly, residents were most likely to spend their time exercising with another resident (58.8%) or with no one (8.8%), although a small proportion of time was also spent with staff (6.8%). Residents were most likely to be stationary when they were on their own (57.3%) or with another resident (34.8%).

**Table 5:** Number of observations, total and mean observation time, and percentage of total observation time by action type and with whom the action was spent with.

What action	Who the action was with	No of observations	Total task time (hours)	Mean observation time (secs)	Percentage of total observation time (%, 95% CI*)
Activity	Family	3	0.5	612.3	0.7 (0–1.4)
	No One	299	61.2	737.2	80.8 (70.6–90.9)
	Other	20	2.8	511.1	3.7 (1.5–6.0)
	Other Resident	114	10.8	342.1	14.3 (10.5–18.1)
	Staff	15	0.4	99.3	0.5 (0–1.2)
Eating	Family	3	0.1	118.3	0.8 (0–3.1)
	No One	20	2.2	401.0	17.9 (9.9–26.1)
	Other	6	0.6	388.7	4.9 (0.1–10.3)
	Other Resident	88	8.6	353.5	69.9 (57.6–82.0)
	Staff	17	0.8	162.4	6.5 (1.3–11.1)
Exercise	No one	10	3.1	1105.2	8.8 (0–24.1)
	Other	5	1.4	1035.6	4.0 (0–11.9)
	Other Resident	127	20.8	590.9	58.8 (50.8–99.7)
	Staff	23	2.4	369.3	6.8 (0–17.0)
Non-observable	Family	10	7.7	2766.0	16.0 (4.4–27.7)
	No One	140	30.4	781.3	63.3 (46.0–80.7)
	Other	4	0.4	383.0	0.8 (0–1.8)
	Other Resident	3	4.1	4888.3	8.5 (0.3–16.7)
	Staff	75	5.4	258.3	11.3 (8.1–14.3)
Sleeping	Family	4	0.1	119.0	0.7 (0–2.8)
	No One	41	12.5	1094.0	88.0 (54.4–100.0)
	Other	1	0.0	24.0	0
	Other Resident	12	1.6	471.0	11.3 (4.6–17.5)
	Staff	8	0.0	17.6	0 (0.1–0.5)
Stationary	Family	13	2.0	545.5	2.2 (0.5–3.9)
	No One	476	51.7	390.8	57.3 (47.8–66.9)
	Other	37	2.6	250.7	2.9 (0–6.0)
	Other Resident	546	31.4	206.9	34.8 (29.9–39.7)
	Staff	132	2.5	68.2	2.8 (1.6–4.0)
Communication	Family	92	8.0	312.6	11.6 (7.7–15.5)
	No One	17	2.1	436.4	3.0 (0.2–5.8)
	Other	118	6.0	181.8	8.7 (5.4–11.9)
	Other Resident	763	37.9	178.8	54.8 (45.7–64.2)
	Staff	890	15.1	61.0	21.9 (19.0–24.7)
Walking	Family	8	0.1	36.0	0.6 (0.3–0.8)
	No One	612	14.1	82.9	90.4 (73.4–100.0)
	Other	7	0.1	67.6	0.6 (0.2–1.4)
	Other Resident	47	0.7	53.4	4.5 (3.0–5.9)
	Staff	39	0.6	57.8	3.8 (1.6–6.4)

*CI = Confidence interval.

### Association of social interactions and quality of life

Residents had a median EQ-5D-5L utility index of 0.68 (IQR = 0.54–0.76). The distribution of responses by the five levels of each EQ-5D-5L dimension indicates that residents often reported having ‘no’ or ‘slight’ problems across all domains (see S1 File). Residents mainly reported having no problems when participating in usual activities in the facility (44%) and in their reports of anxiety and depression (42%). Residents reported severe to extreme difficulties for mobility (24%) and self-care (14%) domains.

**Fig 2:**
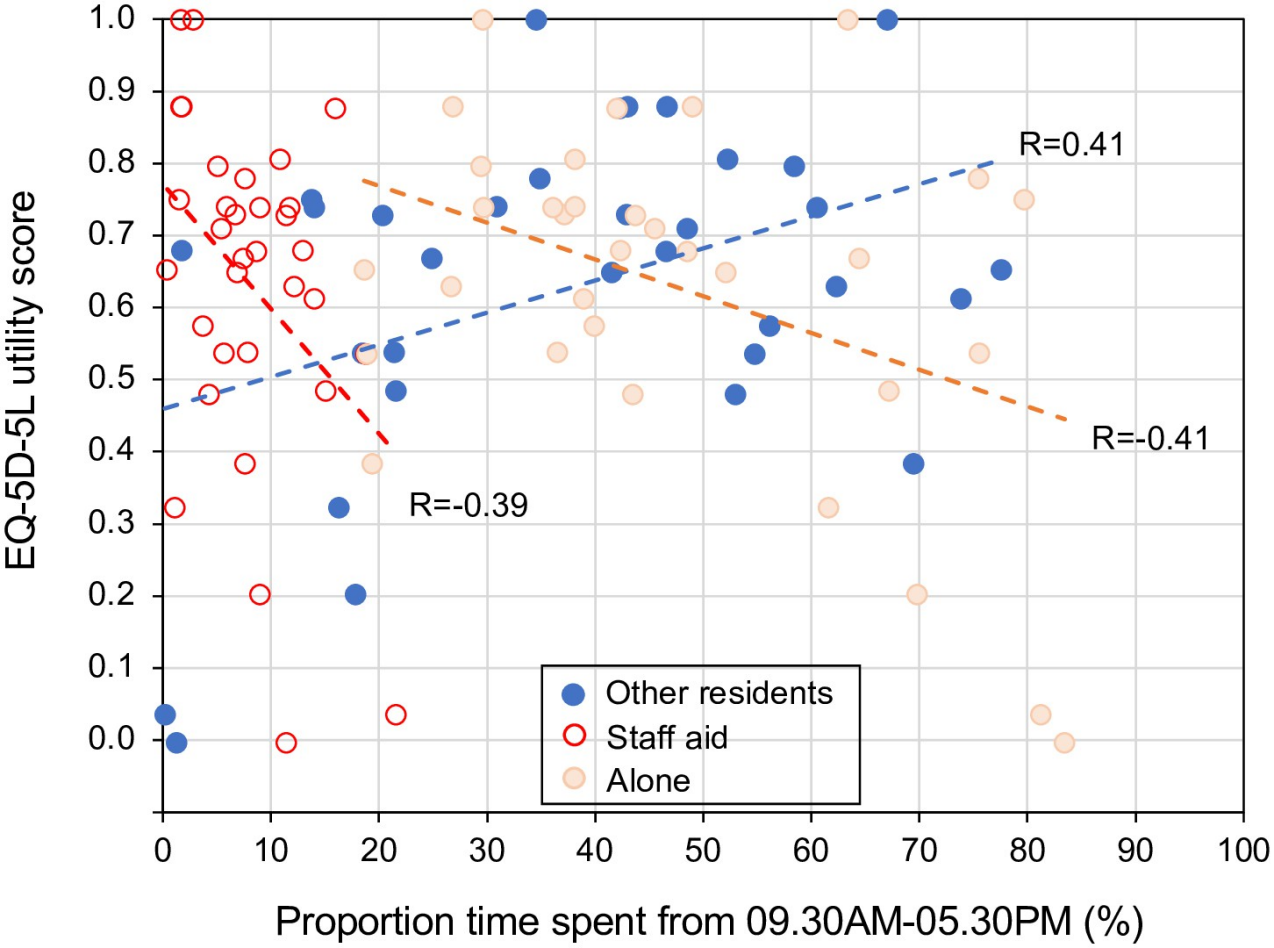
Correlation of quality of life (EQ-5D-5L utility score) and time spent with resident (red) and staff aid (blue).

There was a moderate positive correlation between the proportion of time spent with another resident and a resident’s EQ-5D-5L utility score (r = 0.41, p = 0.018) (Fig 2). There was a moderate negative correlation between the proportion of time spent with a staff member and residents’ EQ-5D-5L utility score (r = -0.39, p = 0.026) and with the proportion of time spent alone and their utility score (r = -0.41, p = 0.017). There were no significant correlations between other factors, including the duration in a location or activities, or frequency of interaction with other individuals, with EQ-5D-5L (p>0.05).

## Discussion

Our results depict a broad summary of resident movement and action patterns in aged care settings and provide a preliminary understanding of the relationship between residents’ social interaction with quality of life. Residents spent time actively in all areas of the facility, including their own room, communal areas and dining room. Residents spent over 50% of their time with other individuals and were engaged in social interactions and activities throughout the day (>52%). Time spent in interpersonal communication was associated with quality of life, suggesting that interactions between residents as well as active participation in organized facility activities could play an important role in maintaining wellbeing.

To the best of our knowledge, this is the first study using observational time-based methodologies to measure social engagement among residents in aged care facilities. The high proportion of time spent in interactive communication (20.2%) and activities (20.5%) observed in this study contrasts with previous international studies [8, 14, 33] where resident inactivity was found to be between 82–97% in the US and 89–92% in Denmark. Our observations further found that residents spent a large proportion of their time alone (47.9%) or with another resident (34.7%). Our findings are similar to a previous Australian study which found residents spent 40% of their time alone, 29% with other residents (29%) and 21% engaged in activities (21%) [9], however differences in concepts of inactivity, measurement methods and focus (e.g., observing care behaviours) limit direct comparisons.

Maintaining social networks after entry into residential aged care is necessary for residents to reinforce their sense of self [34, 35]. Our results found that a considerable proportion of residents’ time was spent with other residents in social dialogue or engaged in activities. This finding could be explained by organisational and environmental influencers. The observed facilities offered multiple scheduled group recreational activities (e.g., arts and crafts, culture clubs, yoga, music therapy, prayer and spiritual reminiscence) which may have contributed to high group attendance. Furthermore, facilities in this study had open-plan layouts, outdoor gardens and practical, easily accessible areas for gatherings, which may promote engagement interactions with other residents [36].

Our results further indicate that residents who spent a larger proportion of their time with other residents reported higher quality of life than those not as engaged with other residents. This result may be partly explained by the influence of peers on individual psychosocial wellbeing. Resident interactions with other residents, whether that be one-on-one or in a group can foster a sense of belonging and purpose [36], which contributes to better wellbeing. Yet, many organisational rules exist as barriers to meaningful interactions and building mutual respect and belonging between staff and residents, and residents with other residents [37, 38]. Regulations around withholding information from residents about other residents (e.g., illnesses and deaths), restricting relationship cultivating practices (e.g. gift exchanges between residents and staff), and frequent staff turnover or shortages often creates an environment unsuitable for nurturing meaningful relationships [37]. Our study suggests that residents, if given the opportunity, may derive significance from their friendships with other residents despite health status.

Similarly, our results found that a higher proportion of time spent with aged care staff was correlated with low quality of life. However, considering that wellbeing and resident care needs are highly related [35–39] and residents with complex care needs often require more comprehensive case management and staff time to assist with their needs [39], our findings are likely a reflection of our residents’ high care needs (requiring almost complete assistance with daily living needs). This confounding variable was unfortunately not able to be controlled for in this study and requires further exploration. Future research should expand on sample size to explore whether other factors such as sociodemographic, mental health, social and family support, and other lifestyle features are associated with quality of life.

A detailed description of residents’ activities throughout the day was a unique aspect of our study. Residents were found to commence the day in either scheduled or self-initiated activities in the common room or own room, which contrasts largely with an earlier study reporting residents sleeping or doing nothing in their own room despite the scheduled morning activities on offer [5]. Our observations during and post lunchtime are aligned with previous findings. We found the dining room became a communal gathering place during lunch and residents returned to their own rooms alone to wait for dinner afterwards [14]. Previous research suggests that many activities in RACFs do not support residents’ personal needs, routines, functional abilities, interests or hobbies, and were partly impeded by unavailable resources [40]. However, our observed increased activity in the morning indicates that residents in the study facilities had opportunities for interaction outside of mealtimes, and that multiple activities were provided for residents.

### Implications

Our results indicate that residents spend a short amount of time with staff that may reflect staff shortages and time pressures which is a long-standing issue in aged care [37]. As such, resident-assisted activities implemented into daily routines could be a useful alternative to generate and strengthen existing resident networks. Employing modified Montessori activities (e.g. in small groups [41], or led by family members [42]) may better suit current staff-resident ratios and promote resident autonomy, limit feelings of boredom, and improve wellbeing. When flexibly tailored these programs can optimise involvement, encourage resident engagement with visitors, improve effect and provide opportunities for meaningful social roles [43].

Additionally, having a higher frequency of tailored activities throughout the day (e.g., morning and afternoon) and adopting flexible personal care schedules could better satisfy psychosocial needs, encourage residents to spend more time with each other, promote positive social interaction and improve feelings of independence and autonomy. Further research into understanding the valued aspects of individual and group resident interactions will offer additional insight into elements that support wellbeing (e.g., presence of greetings, social routines, presence of activity).

### Strengths and limitations

The main strength of the study is the high number of observational hours involved across different aged care facilities. Despite previous studies observing resident activities for a total of 8 to 13hrs, they often used short observational intervals (e.g., 5–10 minutes) [5, 7, 14, 44]. Our work provides a detailed exploration of residents’ daily routines, expanding beyond mealtime practices and resident-staff interactions [45]. We report resident movements and behaviour in real-time and demonstrate that whilst there have been some changes to residents’ daily patterns, rigid traditional activity schedules typical of aged care homes remain somewhat prevalent.

Our study has several limitations. Data were collected during weekdays due to researcher and aged care facility resource constraints which limits when families are likely to visit and is likely to underrepresent the true nature of a residents’ given week [46]. Additional data on clinical outcomes such as incidents, medications, hospital discharge, and clinical deterioration measures (i.e. blood pressure, heart rate) were not collected and may affect resident activity and QoL. Data on subjective QoL and affective responses between individual residents and staff as well as amongst self-reported individual residents, such as feelings of isolation and loneliness were also not collected which may further provide a holistic representation of QoL. Furthermore, selection bias may have been introduced through our recruitment with the aged care facilities. Residents who have comorbidities and disabilities that limit mobility were also not included and this can yield a rarefied sample that is progressively more non-representative robust and healthy.

Finally, the cross-sectional nature of the study prevented a clear definition of the cause and effect of the variables considered. It is unclear whether having low quality of life limits social interactions or vice versa, and correlational analysis has its limitations in drawing conclusions regarding causal relationships amongst variables. Our study, with its small sample size, had insufficient control for potential moderating factors (e.g., greater disability, existing isolation). A larger sample, and longitudinal design, aimed at identifying the specific contribution of each of these factors, are recommended.

## Conclusion

This study describes the observed daily patterns of residents, which compared to the limited available previous research suggest that there is a trend towards greater engagement of residents reflected in time spent in activities and communication outside of residents’ rooms. These findings could be used to tailor interventions to increase opportunities for social interaction, with a focus on flexible, resident-assisted activities. Future studies should adopt a longitudinal design with larger samples to address our current limitations.

## Supporting information

S1 File (DOCX)

## Abbreviations

EQ-5D-5L: Euro-Qol 5 dimension quality of life instrument
QoL: Quality of life
RACFs: Residential aged care facilities
WOMBAT: Work Observation Method By Activity Timing (WOMBAT) software
