# Supplementary material for: Social interactions and quality of life of residents in aged care facilities: A multi-methods study
Source: PLoS One. 2022 Aug 29;17(8):e0273412. doi: 10.1371/journal.pone.0273412 (PMC9423621; doi:10.1371/journal.pone.0273412)
Supplement: S1 File — (DOCX) [file pone.0273412.s001.docx]

**Supplementary Material.**

**Figure S1.** Participant selection flow chart.

**
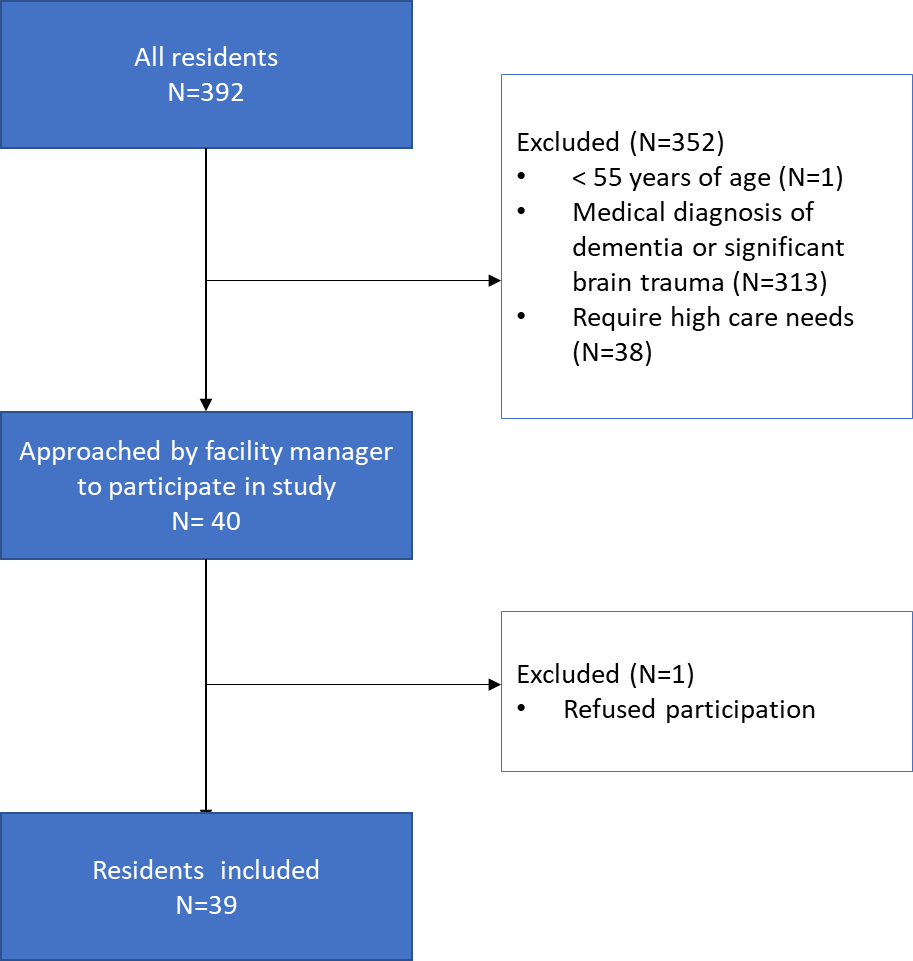
**

**Figure S2.** Screenshot of the WOMBAT program used during observations.


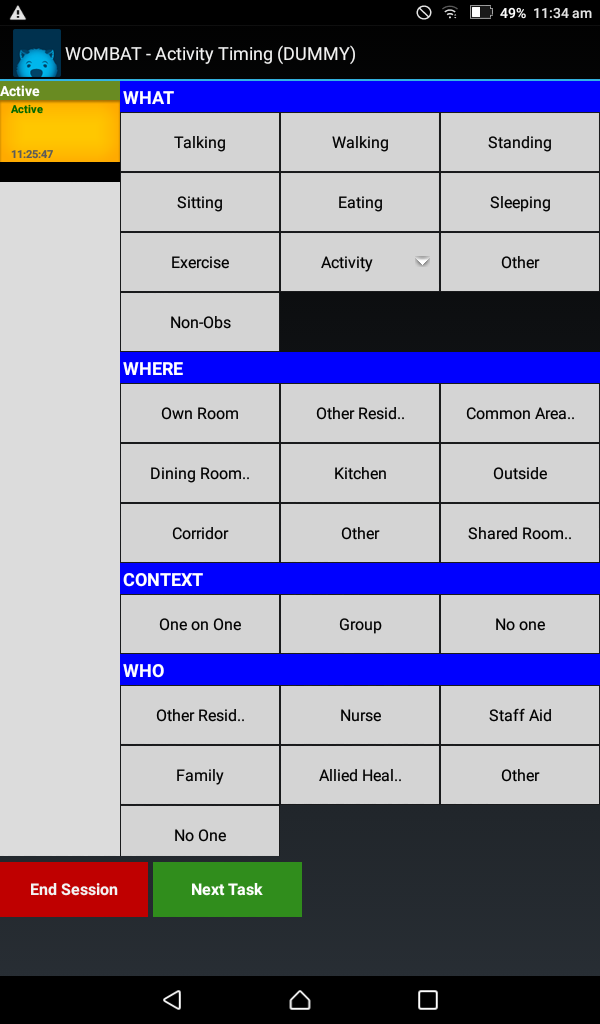

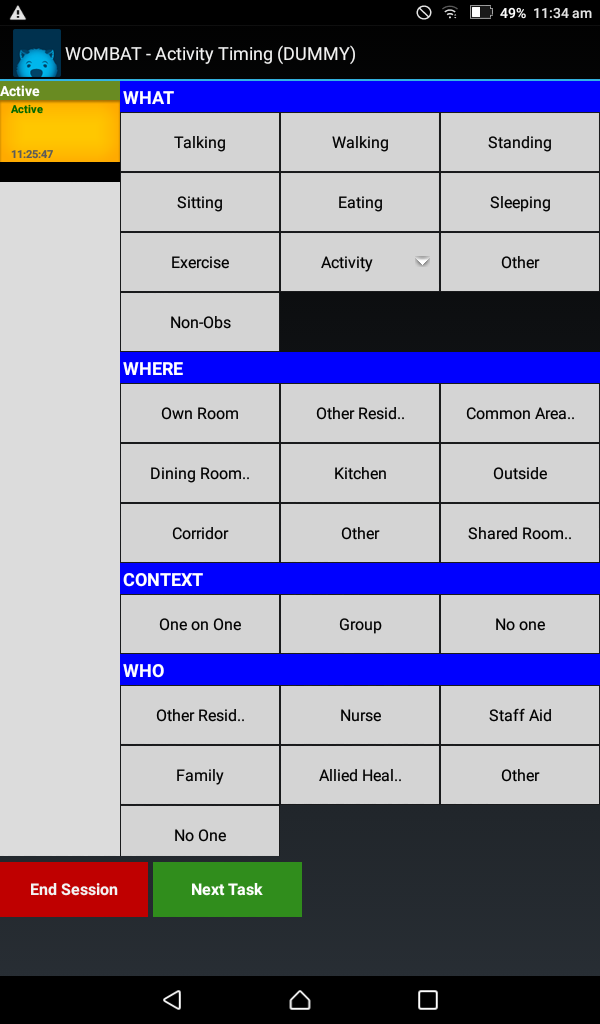


**Figure S3.** Percentage of resident responses (N=39) to each category of the EQ-5D-5L dimensions.

**Table S1.** Sample observation schedule.

| **Date** | | | **1 September** | | | | **2 September** | | | | **3 September** | | | | **4 September** | | | | **5 September** | | | |
| --- | --- | --- | --- | --- | --- | --- | --- | --- | --- | --- | --- | --- | --- | --- | --- | --- | --- | --- | --- | --- | --- | --- |
| **Observer** | | | **A** | | **B** | | **A** | | **B** | | **A** | | **B** | | **A** | | **B** | | **A** | | **B** | |
|  | | | **Resident number** | | | | | | | | | | | | | | | | | | | |
| **Time** | **930am-1130am** | 1 | | 2 | | 3 | | 4 | | 5 | | 6 | | 7 | | 8 | | 9 | | 10 | |  |
|  | **1130am-130pm** | 10 | | 1 | | 2 | | 3 | | 4 | | 5 | | 6 | | 7 | | 8 | | 9 | |  |
|  | **130pm-330pm** | 9 | | 10 | | 1 | | 2 | | 3 | | 4 | | 5 | | 6 | | 7 | | 8 | |  |
|  | **330pm-530pm** | 8 | | 9 | | 10 | | 1 | | 2 | | 3 | | 4 | | 5 | | 6 | | 7 | |  |
